# Impact of Presence, Level, and Closure of a Stoma on Growth in Young Children: A Retrospective Cohort Study

**DOI:** 10.1055/a-2067-4847

**Published:** 2023-05-16

**Authors:** Laurens Donald Eeftinck Schattenkerk, Irene Vogel, Justin R. de Jong, Pieter J. Tanis, Ramon Gorter, Merit Tabbers, L. W. Ernest van Heurn, Gijsbert Musters, Joep P. M. Derikx

**Affiliations:** 1 Department of Pediatric Surgery, Amsterdam UMC Locatie AMC, Amsterdam, the Netherlands; 2 Department of Surgery, Amsterdam UMC Locatie AMC, Amsterdam, the Netherlands; 3 Department of Pediatric Surgery, Emma Children's Hospital, Amsterdam UMC, University of Amsterdam and Vrije Universiteit Amsterdam, Amsterdam, the Netherlands; 4 Department of Surgery, Erasmus MC, Rotterdam, Zuid-Holland, the Netherlands; 5 Department of Pediatric Surgery, Emma Children's Hospital, Amsterdam UMC, Amsterdam, the Netherlands; 6 Department of Pediatric Gastroenterology, Hepatology and Nutrition, Emma Children's Hospital, Amsterdam UMC, University of Amsterdam, Amsterdam, the Netherlands

**Keywords:** surgery, stoma, sodium, supplementation, growth

## Abstract

**Introduction** A stoma will cause nutrients loss which could result in impaired growth. Impaired growth can negatively impact long-term development. This study aims to evaluate: (1) the effect of stomas on growth comparing small bowel stoma versus colostomy and (2) if early closure (within 6 weeks), proximal small bowel stoma (within 50 cm of Treitz), major small bowel resection (≥ 30 cm), or adequate sodium supplementation (urinary level ≤ 30 mmol/L) influences growth.

**Methods** Young children (≤ 3 years) treated with stomas between 1998 and 2018 were retrospectively identified. Growth was measured with weight-for-age Z-scores. Malnourishment was defined using the World Health Organization's definition. Comparison between changes in Z-scores at creation, closure, and a year following closure was done by Friedman's test with post hoc Wilcoxon's signed rank test or Wilcoxon's rank-sum test when necessary.

**Results** In the presence of a stoma in 172 children, 61% showed growth decline. Severe malnourishment was seen at the time of stoma closure in 51% of the patients treated by small bowel stoma and 16% of those treated by colostomy. Within a year following stoma closure, 67% showed a positive growth trend. Having a proximal small bowel stoma and undergoing major small bowel resection led to significantly lower Z-scores at closure. Adequate sodium supplementation and early closure did not lead to significant changes in Z-scores.

**Conclusion** Stomas have a negative impact on growth in the majority of children. This impact might be decreased by preventing small bowel stomas when possible, specifically proximal stomas, and limiting small bowel resection. Since stoma closure is essential in reversing the negative effect on growth, we opt that early closure might result in an early shift to catch-up growth.

## Introduction

In young children (age ≤ 3 years), a small bowel stoma or colostomy might be necessary in the treatment of congenital intestinal diseases or abdominal sepsis. 1 2 Stomas come with a substantial risk of morbidity. Taking into account both stoma creation and closure, major stoma-related morbidity (Clavien–Dindo grade ≥ III) occurs in 39% of the children. 3 Moreover, a stoma increases the loss of fluids and nutrients, which might result in impaired growth. 4 This growth impairment at a young age can negatively impact long-term development and cognitive ability, in particular, when it occurs in the first 9 months of life. 5 6 Moreover, this impairment of growth in itself is associated with an increased risk of stoma-related complications and the need for reoperations. 7 For these reasons, some surgeons recommend early stoma closure, within 6 weeks following formation. 3 4 8 9 10

Children treated by a small bowel stoma are thought to be more prone to growth impairment than children treated by colostomy. The higher risks of a high-output stoma and loss of absorbent function of the small bowel and colon distal of the stoma are suggested to cause this difference between both levels of stoma. 11 12 To what extent such different levels of stoma limit growth in young children is not well known.

Next, type of intestine used to create the stoma, other patient-specific factors, such as proximal small bowel stomas (within 50 cm of Treitz) and undergoing major small bowel resection (≥ 30 cm), might decrease functional bowel length even more, which could further impair growth. Moreover, experimental studies are linking inadequate sodium supply with impaired growth in case of urine sodium levels of 30 mmol/L or less. 13 14 Sodium supplementation might restore the cellular sodium environment and stimulate growth, for which reason this became common practice at many institutions over the past 20 years. However, there are no guidelines on the optimal management of sodium supplementation in the presence of a stoma. 15

Therefore, the aim of this study was to evaluate (1) the effect of constructing either a small or large bowel stoma on growth in young children and (2) the effect of certain patient-specific factors (early stoma closure, having a proximal stoma, undergoing major small bowel resection (≥ 30 cm), or adequate sodium supplementation) on growth status at stoma closure and within a year following closure expressed by weight-for-age Z scores.

## Methods

### Patients and Management

All consecutive young children (≤ 3 years of age) with a small bowel stoma (jejunostomy or ileostomy) or large bowel stoma (colostomy), created between 1998 and 2018 at our tertiary university medical center, were identified from a surgical administrative database. Patients were included in this analysis if (1) they underwent stoma closure and (2) if birth weight, weight at stoma creation and closure, and at least one weight measurement after closure were available. The medical ethical committee of the Academic Medical Center in Amsterdam reviewed and approved the observational study (reference: W18_233#18.278) design. Patients and parents received an opt-out letter for consent. Following consent, patient records were checked for eligibility. Data were retrieved and stored in an electronic database (Castor EDC).

### Data Extraction

We (L.D.E.S. and I.V.) extracted information concerning: gender, prematurity (defined as gestational age < 37 weeks), duration of pregnancy, underlying disease, time to stoma closure (early closure was defined as closure within 6 weeks following creation), need for readmission for stoma closure, need for total parenteral nutrition (TPN) following stoma creation, number of centimeters of small bowel resected (major resection was defined as ≥ 30 cm, independent of age), proximal location of a small bowel stoma (defined as within 50 cm of the ligament of Treitz), weight before stoma creation (weight closest to creation with a maximum of 3 days before creation), weight at stoma closure (weight closest to closure with a maximum of 3 days before or after closure), weight after stoma closure (first measure which was reported in a minimum of 3 months and a maximum of 1 year after closure) and if sodium supplementation was given after stoma creation.

Growth was measured with weight-for-age Z-scores and was calculated with the growth calculator of the Youth Health Department (JGZ). 16 The weight-for-age Z-score (or standard deviation [SD] score) is a measure of the SD for weight from the median value of a reference population matched for duration of pregnancy, age, and sex. 16 The Z-score can be used to discriminate between a child that is gaining weight at a slower (or faster) rate than the reference population and is well suited for a cohort with prematures. 17

Malnourishment was defined according to the World Health Organization's definition; a weight-for-age Z-score between −3 and −2 was classified as mildly malnourished, and a score weight-for-age Z-score below −3 as severely malnourished. 18

The difference between Z-score at stoma creation and closure was calculated to express growth curve in the presence of a stoma, with a negative growth curve indicating growth impairment. The difference between Z-score at stoma closure and Z-score within the year after closure was used to express the growth curve after stoma closure.

Urinary sodium levels were obtained from spot urinary samples obtained after the stoma creation. The current regime at our institute is to evaluate urinary sodium two to three times a week and to start supplementation if urinary sodium is less than 30 mmol/L. Following stoma formation, the lowest urinary sodium measurement for each patient was used defining a urine sodium level of 30 mmol/L or lower as deficient and 10 mmol/L or lower as severely deficient. 14 15 19

There is no official protocol for sodium supplementation at our institute. However, in general practice, 2 mmol/kg oral sodium chloride daily is started, which could be increased with steps of 1 mmol/kg in case of urine sodium levels of 30 mmol/L or lower. If after the start of sodium supplementation, the urinary sodium values were more than twice the levels of 30 mmol/L or lower, the sodium supplementation was defined as inadequate.

### Outcome Measures

The primary outcome was weight-for-age Z-scores. Comparative analyses of the primary outcome were performed between different time points: at the time of creation, closure, and within a year after closure. This was reviewed separately for patients with a small bowel stoma and a colostomy. Differences in Z-scores at closure and within a year following closure were compared between the following groups: (1) early and nonearly closed stomas, (2) proximal and nonproximal small bowel stomas, (3) patients who received a major small bowel resection (larger than 30 cm) and those who did not, and (4) adequate versus nonadequate supplementation of sodium.

### Statistical Analysis

Descriptive data were reported with a median with the interquartile range (IQR). Comparison of changes in Z-scores following stoma closure and a year after closure was done by Friedman's test with post hoc Wilcoxon signed rank test in case of significant results. Comparison between the characteristics of young children with a small bowel stoma and a colostomy was performed with chi-square tests for categorical data, *t* -tests for parametric continuous data, and the Mann–Whitney's *U* tests for nonparametric continuous data. Comparison of changes in Z-scores following stoma closure and a year after closure was done by Wilcoxon's rank-sum test for all secondary outcome measures. Comparison of Z-scores at stoma closure between different groups at closure or at a year following closure was done by Mann–Whitney's *U* tests. All analyses were performed with IBM SPSS statistics, version 23 (IBM Corp., Armonk, New York, United States).

## Results

### Patient Characteristics

A total of 172 young children were included in our analysis. Of all infants, 61% (105/172) were male and the median gestational age was 36 weeks (IQR: 32–38) of which 54% (93/172) were born prematurely. In total, 58% (99/172) of the patients received a small bowel stoma. Of the patients with a small bowel stoma, 10% (10/99) received a jejunostomy and 90% (89/99) an ileostomy. Of all these small bowel stomas, 20% (20/99) were proximal small bowel stomas. The median length of resected small bowel in those treated by ileostomy was 7 cm (IQR: 2–13), and 11% (10/89) received a resection of more than 30 cm. In six (6%) patients treated by a small bowel stoma, data were missing on length of resection. A colostomy was created in 42% (73/172) of the patients. Out of all small bowel stomas, 43% (43/99) were created for treatment of necrotizing enterocolitis. Treatment for an anorectal malformation was the most common (50/73, 63%) reason for a colostomy. Median time to stoma closure was 15 weeks (IQR: 8–30) with a median follow-up of 5 years (IQR: 2–9) after stoma closure. Early closure was performed in 21% (21/99) of the small bowel stomas and 1% (1/73) of the colostomies. A comparison of the patient characteristics of children treated with either small bowel stomas or colostomies is presented in Table 1 .

**Table 1:** Baseline characteristics

Characteristic	Small bowel stoma ( *n* = 99)	Colostomy ( *n* = 73)	*p* -Value
Male gender, *n* (%)	61 (62)	44 (60)	0.89
Premature, *n* (%)	65 (66)	28 (38)	≤ 0.01
Median duration pregnancy (IQR), wk	34 (30–38)	37 (35–39)	≤ 0.01
Underlying disease, *n* (%)			
Anorectal malformation	0	50 (69)	≤ 0.01
Necrotizing enterocolitis	43 (44)	7 (10)	
Hirschsprung's disease	13 (13)	7 (10)	
Intestinal atresia	12 (12)	4 (6)	
Meconium ileus	14 (14)	1 (1)	
Complex gastroschisis	6 (6)	1 (1)	
Other	11 (11)	3 (4)	
Median age at stoma formation (IQR), d	8 (2–17)	3 (1–31)	0.08
Median time to stoma closure (IQR), wk	9 (6.3–15)	28 (18–42)	≤ 0.01
Stoma creation and closure in same admission, *n* (%)	39 (41.5)	1 (1.4)	≤ 0.01
Median time of follow-up (IQR), y	4 (1.25–7)	7 (4–12)	≤ 0.01

Abbreviation: IQR, interquartile range.

### Changes in Z-scores from Birth until a Year after Stoma Closure

**Fig. 1:**
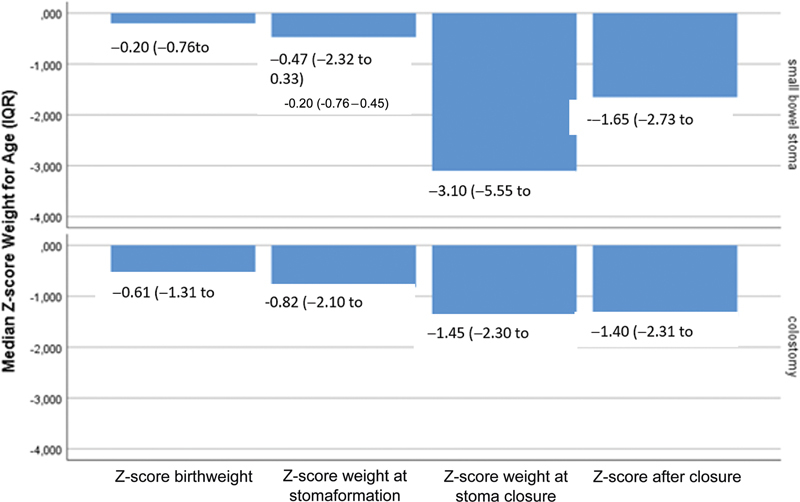
Bar chart median Z-score weight for age (IQR) at birth, stoma formation, stoma closure, and within the year after stoma closure. IQR, interquartile range.

The individual Z-scores for birth weight, weight at the time of stoma creation and closure, and weight measurement within the first year after closure are presented in Fig. 1 and the information on nutritional status and growth are presented in Table 2 . In the presence of a stoma, 61% (105/172) of the young children were declining on the growth chart with a median Z-score for weight for age of −3.1 (IQR: −5.6 to −1.23) in the small bowel stoma group and −1.45 (IQR: −2.3 to −0.62) in the colostomy group at the time of stoma closure. This resulted in severe malnourishment during the stoma closure in 51% (50/99) of the patients with a small bowel stoma, and 16% (12/73) of those with a colostomy. Median time to stoma closure was 9 weeks (IQR: 6.3–15) in patients treated by a small bowel stoma and 28 weeks (IQR: 18–42) in the case of a colostomy. After stoma closure, most young children were thriving with a positive tract on the growth chart, 74% (73/99) versus 58% (42/73) in the small bowel stoma and colostomy groups, respectively.

**Table 2:** Comparison of nutritional status and growth in infants with a small bowel stoma and colostomy

Characteristic	Small bowel stoma ( *N* = 99)	Colostomy ( *N* = 73)	*p* -Value
TPN after stoma creation, *n* (%)	74 (76)	24 (37)	≤ 0.01
Sodium supplementation after stoma creation, *n* (%) a	71 (86)	24 (49)	≤ 0.01
TPN poststoma closure, *n* (%)	57 (62)	4 (6)	≤ 0.01
Nutrition status at the time of stoma closure, *n* (%)			
Normal	38 (38)	49 (67)	≤ 0.01
Malnourished	11 (11)	12 (16)	
Severely malnourished	50 (51)	12 (16)	
Positive growth tract presence stoma, *n* (%)	40 (40)	27 (40)	0.65
Positive growth tract after stoma closure, *n* (%)	73 (74)	42 (58)	0.03

Abbreviation: TPN, total parenteral nutrition.

a Sodium supplementation unknown small bowel stoma *n* = 16, colostomy *n* = 24.

The change in Z-score from the time point of stoma formation to stoma closure and to the time point within 3 months to a year after closure (mean: 9.2 months, SD: 5 months) was significant in both patient groups treated by small bowel stoma ( *p* < 0.00) as well as colostomies ( *p* = 0.04). Post hoc test showed that within small bowel stomas, this difference in Z-score was most profound between stoma formation and stoma closure ( *p* < 0.00) showing a decline in Z-score as well as the moment of stoma closure and within a year following closure ( *p* < 0.00) showing an incline. Z-scores did not differ significantly between moment of stoma formation and within a year following closure ( *p* = 0.44). In colostomies, the post hoc test showed a borderline nonsignificant ( *p* = 0.07) difference in Z-scores between the moment of stoma closure and within a year following closure. The differences in Z-scores between formation and closure ( *p* = 0.10) and formation and within a year following closure ( *p* = 0.24) were similar.

### Factors Influencing Growth in Patients Treated with a Stoma

There was no significant difference in Z-score at the moment of closure ( *p* = 0.19) as well as the Z-score within the year after closure ( *p* = 0.20) when comparing patients who had early closure and those who had not. The age at closure differed significantly ( *p* < 0.00) between those who had an early closure (median 11 months; IQR: 9–14) and those who had not (median: 16 months; IQR: 11–21).

Those treated with a proximal small bowel stoma had significantly lower Z-scores ( *p* = 0.01) at stoma closure compared with those with a nonproximal small bowel stoma, but weight within a year after closure did not differ between both groups ( *p* = 0.07). Moreover, those who received a major small bowel resection had a lower Z-score at stoma closure ( *p* = 0.04) than those with less than 30 cm of resected small bowel. This significant difference disappeared within a year after closure at which moment the Z-scores did not differ between the groups ( *p* = 0.27).

From the 172 young children, 49% (84/172) had data regarding urinary sodium measurements. After the creation of a small bowel stoma, 92% (55/60) of the young children had a mild or severe sodium deficiency, following a colostomy, this was 83% (20/24) ( Table 3 ). Of the young children with a small bowel stoma, 92% (55/60) received sodium supplementation. In 68% (41/60) of patients, there were more than two urine sodium levels less than 30 mmol/L after the start of supplementation, indicating inadequate sodium supplementation. In the patients with a colostomy, 54% (13/24) received sodium supplementation which was inadequate in 29% (7/24). The median Z-score at stoma closure of adequately supplemented children was −2.4 (IQR: −4.3 to −1.3) which is lower than the median in nonadequately supplemented children which was −1.7 (IQR: −3.4 to −0.5). This difference was borderline nonsignificant ( *p* = 0.06). A year after closure, this difference remained nonsignificant ( *p* = 0.42). Also, there was no correlation ( *p* = 0.50) between adequate sodium supplementation and a positive growth curve in the presence of a stoma.

**Table 3:** Comparison of urinary sodium levels after creation of a small bowel stoma and colostomy and the effect of sodium supplementation

Characteristic	Small bowel stoma ( *n* = 60)	Colostomy ( *n* = 24)
Lowest urine sodium level, mmol/L		
> 30	5 (8)	4 (17)
10–30	31 (52)	11 (46)
< 10	24 (40)	9 (37)
Sodium supplementation		
Adequate	14 (24)	6 (25)
Inadequate	41 (68)	7 (29)
None	5 (8)	11 (46)

## Discussion

In the presence of a stoma, the majority (61%) of the patients were declining on the growth chart. This resulted in severe malnourishment in 51% of the young children with a small bowel stoma and in 16% of the patients with a colostomy at the time of stoma closure. After stoma closure, the decline in Z-scores is reversed in most young children; 67% showed a positive trend on the growth chart within a year following stoma closure. The decline in growth during the treatment with a stoma is more profound in small bowel stomas compared with colostomies. Growth at stoma closure was significantly more impaired in those treated with a proximal small bowel stoma and those who received a stoma after major small bowel resection. Early closure did not significantly affect Z-scores at stoma closure, although closure is realized at a significantly younger age. Within a year following closure, none of the evaluated factors had a significant influence on the Z-scores.

Our results are in line with previous reports in small cohorts of infants treated with a stoma for multiple abdominal diseases and show that in the presence of both small and large bowel stomas, a decline in growth can be expected. 14 Those treated with a small bowel stoma and specifically those treated with a proximal small bowel stoma or those undergoing major small bowel resection seem most at risk. Stoma excretion from the small bowel is higher in nutrients than excretion from colostomies which suggests that in the latter, more nutrients are resorbed which might contribute to this difference in growth. 20 This could also explain why more patients treated with a small bowel stoma were in need of TPN following stoma formation compared with colostomies. This difference in functional proximal bowel might also explain why patients with more proximal stomas and those who underwent a major resection have significant lower Z-scores at stoma closure. Another explanation could be found in the differences in the types of disease which result in small bowel stomas, in our cohort mostly necrotizing enterocolitis. In patients treated for necrotizing enterocolitis, length of resected intestine and prolonged inflammation both negatively influence growth. 8 21

It seems that stoma closure as soon as possible is a necessity for growth in all young patients which is in line with previous findings. 4 Even those at highest risk of growth impairment at the moment of stoma closure (patients treated by proximal ileostomy and those who underwent a major small bowel resection) show similar growth within a year following closure compared with patients with nonproximal small bowel stomas and those who did not underwent a major resection. This suggests that patients experience catch-up growth following stoma closure, even when there is less functional small bowel left, either due to resection or due to underdevelopment caused by disuse. Since stoma closure seems such an important condition for growth, this could be seen as an argument for early closure. In young children, there is no consensus on the optimal timing of stoma closure. Some surgeons would wait for a safe weight (e.g., > 2.5 kg) to reduce the risk of surgery in a fragile patient. 22 Other, more recent studies report no significant difference in postoperative complications when a stoma is closed early (within 6–8 weeks), even with a low body weight. 18 23 An argument against early closure is the assumed risk of adhesions which might result in a difficult operation. However, in patients treated for necrotizing enterocolitis, there was no difference in the presence of adhesions between early and late closure of stomas. 24 Within our own cohort, we could not provide evidence that early closure may also lead to higher Z-scores at closure compared with nonearly closure. A reason could be that early closure in our cohort was mostly performed due to stoma complications, such as high-output or repeated prolapses, which might themselves have negatively influenced growth. Still, we showed that early closure results in the same amount of catch-up growth within a year following closure as in those nonearly closure. Since early closure results in a significantly lower age at closure compared with nonearly closure, we can at least say that it seems that early closure results in an early shift to catch-up growth gaining weeks of advantage.

Besides growth impairment, other complications after stoma creation can occur such as surgical site infections and high-output stomas. 3 Moreover, closing the stoma also leads to both short-term complications, such as anastomotic leakage, and long-term morbidity, such as adhesion-related small bowel obstruction and incisional hernia. 12 25 Taking into account the high risk of complications and the risk of growth impairment, one might consider performing primary anastomosis instead of stoma creation with a lower threshold. Primary anastomosis has been shown to be feasible in selected patients treated for necrotizing enterocolitis or intestinal atresia. 12 26 27 Some situations, such as bowel perforation or meconium peritonitis, might necessitate stoma creation, but the associated risk of morbidity should be taken into account when deciding on whether or not to create a stoma.

In the presence of a stoma, oral sodium supplementation has been reported to improve weight gain. 14 15 28 Supplementation will counter the loss of sodium, which is partly excreted via stoma production, predominantly in small bowel stomas. However, sodium is also lost via renal excretion which is most prominent in premature born children. 29 This might explain why patients with a colostomy in this study were sometimes also found to be sodium depleted. Both the diagnosis and risk of sodium depletion in young children with a stoma are poorly understood. However, young children with a low urine sodium concentration (< 30 mmol/L) have been shown to gain significantly less weight than those with normal urine sodium levels. 19 We could not verify these results in our cohort. There are currently no guidelines for correct sodium supplementation in young children with a stoma, and there are only small reports with suggested treatment protocols specifically for premature born neonates. 15 The lack of sufficient sodium supplementation in our cohort is indicating the need for a clearly defined protocol for oral sodium supplementation in young children with a stoma. An important part of such a protocol is how to evaluate the true body sodium levels and what substrate to use. There are multiple possibilities opted, the best method of which is suggested to be a 24-hour urine collection. 15 This method is often too burdensome for young children who recently underwent surgery, and insertion of a urinary catheter would be required. Another suggested option is to make use of serum sodium. 15 However, venipuncture for diagnosis is the primary cause for neonatal anemia, and therefore, regular determination of serum sodium is not recommended. 30 In practice, urinary sodium concentration measured from a spot urine sample is an acceptable, noninvasive, and inexpensive method. Still, the question is what level of spot urine sodium reflects true sodium deficiency. It could be that a change in definitions, for instance only defining inadequate supplementation after three measurements of 30 mmol/L or lower, would prove a better reflection of the true sodium levels. This could explain why we could not find a correlation between adequate supplementation and positive growth and why it seemed that adequately supplemented children showed a trend toward lower Z-scores at closure than nonadequately supplemented children, although other explanations, such as delayed growth, might apply.

Limitations of this study are the retrospective design which resulted in exclusion of a proportion of our cohort due to missing data regarding Z-scores. This might have led to selection bias, for instance due to over/underinclusion of certain types of diseases, and possibly type II errors, for instance in the evaluation of patients receiving early closure. Also, patients were not randomly assigned to receive certain treatments, such as early closure or major small bowel resection. This could have resulted in allocation bias, possibly influenced by factors such as disease severity or occurrence of stoma-related complications. Moreover, weight measurement was all single measurement which could vary from day to day. Growth is a complex process affected directly or indirectly by a multitude of interrelated factors which is why it is hard to determine the exact etiology of growth impairment. Our results seem to show that growth decreases in the presence of a stoma, which in most patients is only reversed after stoma closure. It could be that other confounding factors, for instance diet, might explain at least some of these changes in growth. Finally, due to the retrospective nature of this study, we were limited in the factors we could retrieve. There are, for instance, other manners of assessing nutritional status of patients such as weight for height and middle upper arm circumference and other factors that could influence weight such as fluid balance. We also could not retrieve information on refeeding, which has been opted to have a positive effect on growth in neonates, specifically prematures with low birth weight, treated with an intestinal stoma. 31

## Conclusion

A weighted decision on the creation of a stoma and timely stoma closure is important, considering the negative impact that a stoma has on growth in the majority of children. This impact might be decreased by preventing small bowel stomas when possible, specifically those more proximal than 50 cm before Treitz, and limiting small bowel resection. Moreover, most young children with a stoma do receive sodium supplementation, but there is no guidance on optimal supplementation and in a large proportion the supplementation seems to be inadequate. Since stoma closure seems essential in reversing the negative effect seen in most patients with a stoma, we opt that early closure, especially in children treated by small bowel stomas, results in an early shift to catch-up growth. Since there are currently no guidelines focusing on the moment of stoma reversal as well as sodium supplementation and monitoring in these patients, this demands future attention.
